# Deep learning-based insights on T:R ratio behaviour during prolonged screening for S-ICD eligibility

**DOI:** 10.1007/s10840-022-01245-6

**Published:** 2022-05-13

**Authors:** Mohamed ElRefai, Mohamed Abouelasaad, Benedict M. Wiles, Anthony J. Dunn, Stefano Coniglio, Alain B. Zemkoho, Paul R. Roberts

**Affiliations:** 1 Cardiac Rhythm Management Research Department, University Hospital Southampton NHS Foundation Trust, Southampton, UK; 2 Faculty of Medicine, University of Southampton, Southampton, UK; 3 Cardiology Department, King’s College Hospital NHS Foundation Trust, London, UK; 4 School of Mathematical Sciences, University of Southampton, Southampton, UK

**Keywords:** Subcutaneous implantable cardiac defibrillator, Deep learning methods, Artificial intelligence, Cardiac devices

## Abstract

**Background:**

A major predictor of eligibility of subcutaneous implantable cardiac defibrillators (S-ICD) is the T:R ratio. The eligibility cut-off of the T:R ratio incorporates a safety margin to accommodate for fluctuations of ECG signal amplitudes. We introduce a deep learning-based tool that accurately measures the degree of T:R ratio fluctuations and explore its role in S-ICD screening.

**Methods:**

Patients were fitted with Holters for 24 h to record their S-ICD vectors. Our tool was used to assess the T:R ratio over the duration of the recordings. Multiple T:R ratio cut-off values were applied, identifying patients at high risk of T-wave oversensing (TWO) at each of the proposed values. The purpose of our study is to identify the ratio that recognises patients at high risk of TWO while not inappropriately excluding true S-ICD candidates.

**Results:**

Thirty-seven patients (age 54.5 + / − 21.3 years, 64.8% male) were recruited. Fourteen patients had heart-failure, 7 hypertrophic cardiomyopathy, 7 had normal hearts, 6 had congenital heart disease, and 3 had prior inappropriate S-ICD shocks due to TWO. 54% of patients passed the screening at a T: R of 1:3. All patients passed the screening at a T: R of 1:1. The only subgroup to wholly pass the screening utilising all the proposed ratios are the participants with normal hearts.

**Conclusion:**

We propose adopting prolonged screening to select patients eligible for S-ICD with low probability of TWO and inappropriate shocks. The appropriate T:R ratio likely lies between 1:3 and 1:1. Further studies are required to identify the optimal screening thresholds.

## Introduction

The subcutaneous implantable cardiac defibrillator (S-ICD) was designed to offer defibrillation protection while avoiding lead-related complications associated with traditional ICDs. However, the consequence of S-ICD programming is a risk of T-wave over sensing (TWO). The eligibility for S-ICD is identified during a mandatory screening process where surface ECGs done in multiple postures are used as surrogates of S-ICD vectors. The ECGs are then analysed against acceptable templates — now largely done through an automated screening tool — to determine eligibility. At least one vector needs to pass screening in at least two postural positions for the patient to be deemed eligible. A major predictor of eligibility of a vector is the T:R ratio. Tools in current screening practice recommended by the manufacturer propose a T:R ratio of 1:3 as a cut-off for S-ICD eligibility. Inappropriate S-ICD shocks due to TWO remain an issue despite current screening practices [1].

The cut-off T:R ratio of 1:3 used for current screening practice incorporates a safety margin to accommodate for the fluctuations of the ECG signal amplitudes over time without affecting the sensing of the S-ICD. Based on a deep learning method developed by some of the authors in this study [2], we conduct a prolonged screening for S-ICD capable of accurately measuring the degree of the T:R ratio fluctuation over the monitoring/screening period. Crucially, the tool can help identify patients with high probability of TWO and inappropriate shocks that can be missed using the current screening practice.

However, utilising a T:R ratio of 1:3 for prolonged screening is likely to unnecessarily exclude a significant number of patients who are otherwise appropriate candidates for S-ICD therapy. Attempting to find the appropriate ratio that identifies patients who are at high risk of TWO and inappropriate shocks while not inappropriately excluding true S-ICD candidates after prolonged screening is uncharted territory. The purpose of this study is to provide groundwork for future trials to answer this question.

## Methods

This is a prospective observational study on a mixed cohort of patients which is divided into 5 subgroups:

1. Patients with a recorded diagnosis of the clinical syndrome of heart failure and receiving intravenous diuretic therapy (at least 120 mg furosemide/24 h) on clinical grounds under the discretion of their treating physicians.
2. ACHD patients.
3. Adult patients with HCM.
4. Adult individuals who have no known structural heart disease for the healthy volunteer’s subgroup.
5. Adult patients who have had previous documented inappropriate shock(s) by their S-ICDs attributed to TWO.

Patients’ demographics were obtained from the medical records. There was no requirement for further patient visits or follow-ups. The study was performed with approval from the REC (17/SC/0623) and was granted R&D (RHMCAR0528) approval.

**Fig. 1:**
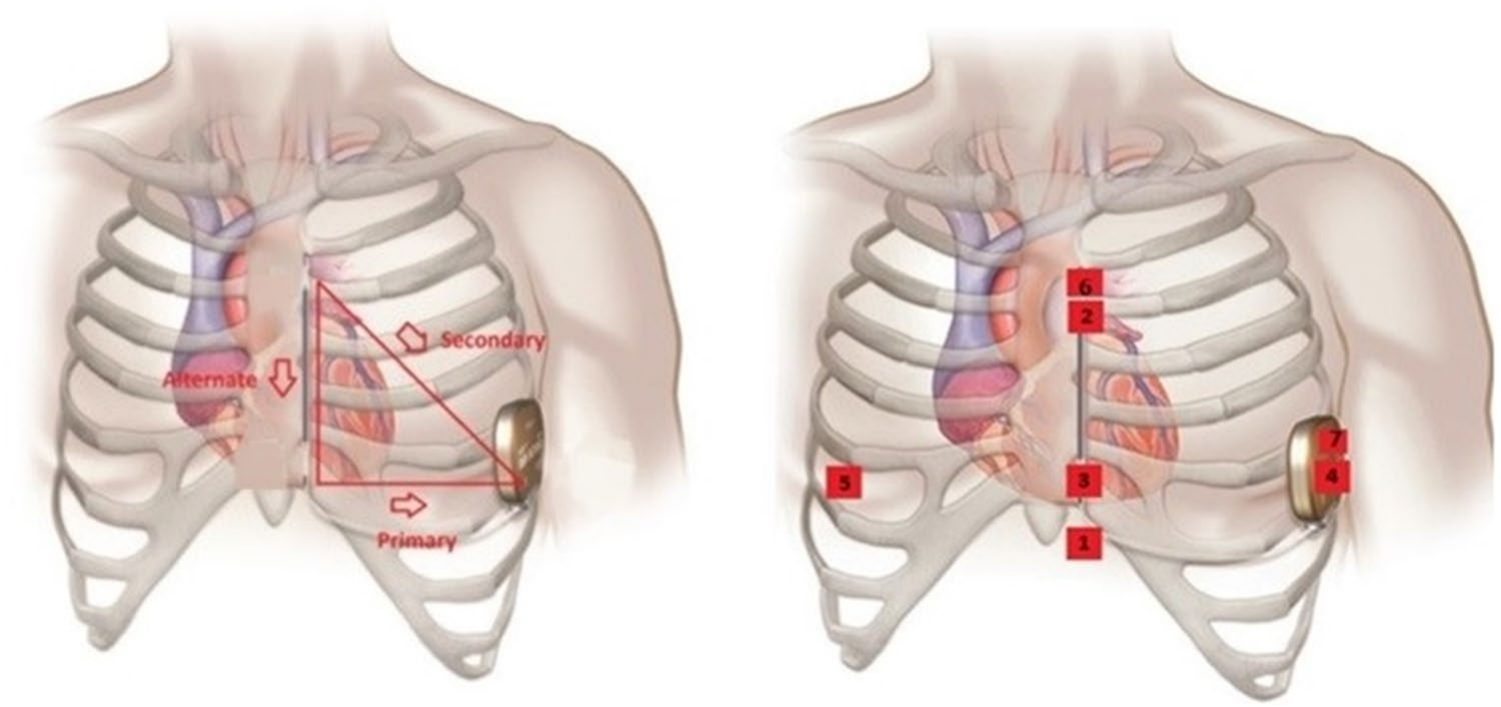
The typical S-ICD vectors on the left and on the right, the Holter® surface ECG positions. 1 = 1 cm infero-lateral to the xiphisternum; 2 = 14 cm superior to position 1; 3 = 5th intercostal space, parasternal position; 4 = 6th intercostal space left mid axillary line; 6 = adjacent to 2; 7 = adjacent to 4. Holter channel A records between points 1 and 4 = surrogate of S-ICD primary vector; Holter channel B records between points 2 and 3 = surrogate of S-ICD alternate vector; Holter xhannel C records between points 6 and 7 = surrogate of S-ICD secondary vector; 5 = 5th intercostal space right mid clavicular line = neutral electrode. Image prior to annotation © Boston Scientific Corporation or its affiliates

All the participants were asked to wear a seven lead/ three channel Holter monitors for 24 h. The leads for the Holters were positioned so that they mimic and correspond to the three vectors (primary, alternate, and secondary) of an S-ICD (Fig. 1). The T:R ratio was monitored throughout 24 h due to it being one of the main determinants of S-ICD eligibility.

### Utilisation of artificial intelligence

Machine learning methods are already being used in the classification and the prediction of various cardiovascular diseases through ECG data analysis [3–11]. Neural networks have been used before in ECG analysis for classifying heart attacks and arrhythmias, as well as for predicting blood pressure [3–5, 10–14]. A well-recognised technique for processing ECG data is to create its phase space reconstruction (PSR) matrix, a popular technique in waveform analysis for representing nonlinear characteristics of time series set of data using delay maps.

The tool developed by some of the authors of this paper [2] and specifically designed for the purpose of this study was used to track and analyse the T:R ratios for the leads corresponding to the S-ICD vectors over the 24-h recordings efficiently and accurately. Raw data from the Holters were first downloaded in ASCII format at a frequency of 500 Hertz (Hz), then split into 10-s segments. Baseline drift correction techniques were then applied, followed by adaptive band stop filtering to suppress power-line noise with a frequency of 50 Hz while a low pass filter (40 Hz) was used to remove the remaining high frequency noise. Each 10-s segment of ECG data is then transformed into a single 32 × 32 pixel image of its PSR matrix. A deep learning model was trained to predict the T:R ratio from these PSR images with a high degree of accuracy. The end result is a plot showing the variation of the T:R ratios for each lead/S-ICD vector over the recorded period [2].

### Deep learning tool validation and accuracy

The deep learning tool was trained using tenfold cross validation. ECG segments with pre-determined manually measured T:R ratios were used to train the tool, while a proportion of the segments were blinded from the tool and were subsequently used for a series of experiments to assess the tool for accuracy. The outcome of the tool (predicted T:R values) was compared to the previously manually measured T:R values. Several standard accuracy parameters were used to assess the accuracy of the tool; mean squared error (MSE) = 0.0122, root mean squared error (RMSE) = 0.0938, and mean absolute error (MAE) = 0.046. Having an MAE of 0.046 means that on average, the difference between the tool-predicted T:R ratio and the manually measured T:R was 0.046. The results of these accuracy parameters were very favourable denoting high level of accuracy for our tool. We invite readers further interested into the details of the mathematical aspects of our deep learning tool to refer to the previously published work by Dunn et al [2].

The tool [2] is used to give the T:R ratio for every 10 s of data/ECG signals (equivalent to a standard 12-lead ECG or a standard ECG strip used for current S-ICD screening process [15]); this allowed the assessment of the S-ICD screening eligibility for every 10 s for the whole 24-h screening. From there, the probability of each patient passing the screening using the current screening practices if they had their screening done at any time of the day is calculated. The probability equals the number of 10-s segments where at least one Holter lead/S-ICD vector exhibited favourable (< 1:3) T:R ratio divided by the total number of 10-s segments (8640) in a 24-h recording.

For the proposed prolonged screening methodology, a time interval of 20 consecutive seconds of unfavourable T:R ratio was chosen as a cut off for failing the screening. This is based on the detection, charge, and redetection time of the current S-ICD system. In other words, a TWO episode has to be at least this long for the S-ICD system to over sense the T-waves, mistakenly interpret the presenting rhythm as ventricular tachycardia, charge the can, confirm the underlying rhythm, then deliver a shock — an inappropriate one in this case. For the sake of the study, the delivery of a hypothetical inappropriate shock was chosen as a hard cut off criterion for failing the S-ICD screening. It can appear that the S-ICD screening criteria for this proposed methodology is too lax, allowing leads/vectors exhibiting “silent” TWO episodes to pass the screening. However, these proposed criteria are meant to provide a reference point to compare the different S-ICD vectors results against, as a proof of concept. In real life screening, the tool could be adjusted for T:R thresholds as well as time intervals if needed.

Multiple T:R ratio cut-offs were then applied on the whole duration of the recordings (24 h), identifying the proportion of patients that would be deemed at high risk of inappropriate shocks due to TWO at each of the proposed cut off values (1:3, 1:2, 2:3, 3:4, and 1:1) for the T:R ratio. For a patient to pass the screening, they would have to have at least one vector eligible at the proposed T:R threshold throughout.

### Statistical methods

Data analysis was done using RStudio 1.4.1106 running R 4.0.5. The categorical data were represented as n/N (%) and continuous data as mean (SD). Fisher’s exact test was used to analyse the contingency tables given the small sample size and compared continuous non-parametric data using Kruskal–Wallis rank sum test between the different studied groups.

## Results

A total of 37 patients were included in the study. The mean age of our participants is 54 ± 21.3 years, 24 males (64.8%). The study population presents a mixed cohort of underlying aetiologies; 14 patients had heart failure (HF), 7 HCM, 7 had apparently normal hearts, 6 had adult congenital heart disease (ACHD), and 3 patients had received inappropriate shocks via their S-ICDs in the past due to TWO, see Table 1 for participants demographics.

**Table 1:** Patients’ demographics

Total number of participants		*n* = 37	
Demographics:	Mean age (years ± 95% *CI*)	54.5 ± 21.3	
	Male	24	64.8%
Cardiac co-morbidities:	Heart failure	14	37.8%
	Atrial fibrillation	7	18.9%
	LV diastolic dysfunction	5	13.5%
	Ischaemic heart disease	8	21.6%
	LV systolic dysfunction	14	37.8%
	Hypertrophic cardiomyopathy	7	18.9%
	Adult congenital heart disease	7	18.9%
	S-ICD in situ	3	8.1%
	Apparently normal hearts	7	18.9%

The overall probability of the patient cohort to have passed the screening using the standard screening methods used in current practice based on a T:R ratio of 1:3 is 0.96 ± 0.13 regardless of underlying aetiology. The probabilities are 0.83 ± 27, 1.0, 0.97 ± 0.1, and 1.0 for the ACHD, HCM, HF, and the normal heart subgroups respectively. Interestingly, the cohort of the three patients who had previously experienced inappropriate S-ICD shocks due to TWO also had a very high probability of passing the standard S-ICD screening (0.99 ± 0.01) (*p* = 0.02), see Table 2 for detailed results.

**Table 2:** Probability of passing standard S-ICD screening in a 24-h period based on a T:R of 1:3

Characteristic	Overall, *N* = 37^1^	ACHD, *N* = 6^1^	HCM, *N* = 7^1^	HF, *N* = 14^1^	Normal, *N* = 7^1^	S-ICD, *N* = 3^1^	*p*-value^*2*^
Outcome	96 (13)%	83 (27)%	100 (0)%	97 (10)%	100 (0)%	99 (1)%	**0.02**
Age	55 (21)	39 (20)	57 (25)	70 (11)	36 (8)	53 (28)	**0.005**
Gender							0.62
F	13/37 (35%)	1/6 (17%)	3/7 (43%)	4/14 (29%)	4/7 (57%)	1/3 (33%)	
M	24/37 (65%)	5/6 (83%)	4/7 (57%)	10/14 (71%)	3/7 (43%)	2/3 (67%)	

^1^Mean (SD); *n*/*N* (%). ^2^Kruskal-Wallis rank sum test; Fisher’s exact test

It is important to highlight that this probability of passing the screening is calculated based upon the current screening practice of having at least a single vector, at any point of time that meets the screening criteria for the short duration of time when the screening is being performed. It is a hypothetical scenario to illustrate the likelihood/probability that a patient would have been deemed S-ICD eligible if this patient had their screening done utilising the current/one point in time screening methods at any time within 24 h.

These high probabilities can be misleading. One would suspect that the dynamicity of the T:R ratios would make a participant pass at one time in a certain vector and at another in different time; this makes the high probabilities less meaningful, as S-ICDs can only be programmed at a fixed vector at one time and do not automatically change them.

**Fig. 2:**
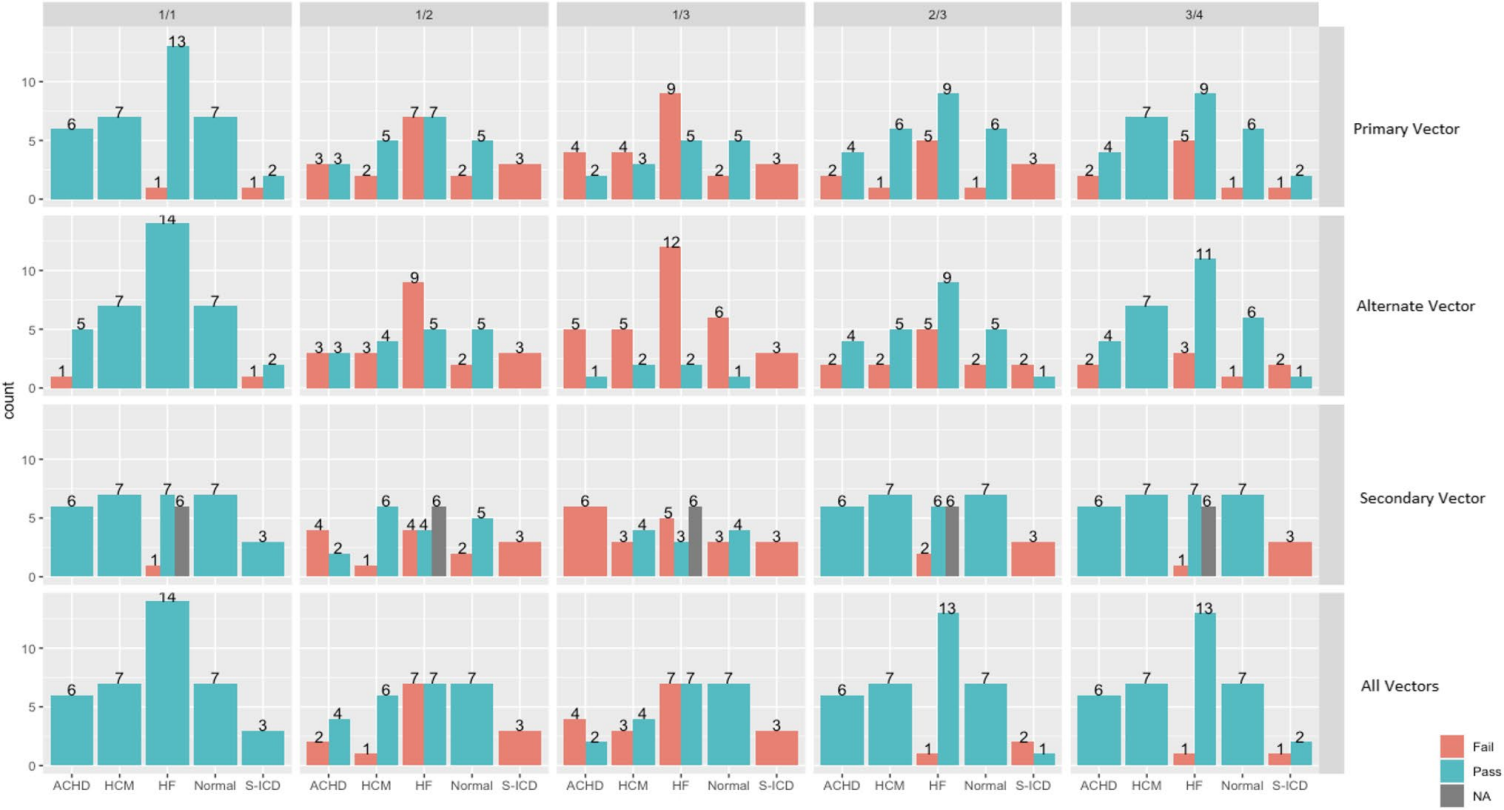
The S-ICD vectors screening pass rates for each S-ICD vector, for all our proposed thresholds and for all the underlying aetiologies that were represented in our analysis

Since S-ICDs can only be programmed at one fixed vector at a time, for our proposed prolonged screening method — using our tool — we followed each vector separately throughout the recordings in all the participants, and then based on the pre-defined thresholds, as explained in the methodology section, each vector would be deemed to have passed/failed the screening independently from the other two vectors of the same participant. A patient is deemed to have passed the screening if they exhibited at least one vector that passed the screening throughout. Only 20 (54%) patients — regardless of the underlying aetiology — would have passed the screening if the same T:R ratio of 1:3 was utilised for prolonged screening. All the patients would have passed the screening if a T:R ratio of 1:1 was utilised for prolonged screening. For T:R ratios of 1:2, 2:3, and 3:4, 65%, 92%, and 95% of the patients would have passed the screening, respectively, see Tables 3, 4, 5, 6, 7, and 8 and Fig. 2 for detailed results.

**Table 3:** S-ICD screening success rates at different thresholds for all patients

Threshold/subgroup	Overall, *N* = 37^1^	ACHD, *N* = 6^1^	HCM, *N* = 7^1^	HF, *N* = 14^1^	Normal, *N* = 7^1^	S-ICD, *N* = 3^1^	*p*-value^2^
T:R threshold 1:3 (0.33)							0.022
Fail	17/37 (46%)	4/6 (67%)	3/7 (43%)	7/14 (50%)	0/7 (0%)	3/3 (100%)	
Pass	20/37 (54%)	2/6 (33%)	4/7 (57%)	7/14 (50%)	7/7 (100%)	0/3 (0%)	
T:R threshold 1:2 (0.5)							0.013
Fail	13/37 (35%)	2/6 (33%)	1/7 (14%)	7/14 (50%)	0/7 (0%)	3/3 (100%)	
Pass	24/37 (65%)	4/6 (67%)	6/7 (86%)	7/14 (50%)	7/7 (100%)	0/3 (0%)	
T:R threshold 2:3 (0.66)							0.025
Fail	3/37 (8.1%)	0/6 (0%)	0/7 (0%)	1/14 (7.1%)	0/7 (0%)	2/3 (67%)	
Pass	34/37 (92%)	6/6 (100%)	7/7 (100%)	13/14 (93%)	7/7 (100%)	1/3 (33%)	
T:R threshold 3:4 (0.75)							0.37
Fail	2/37 (5.4%)	0/6 (0%)	0/7 (0%)	1/14 (7.1%)	0/7 (0%)	1/3 (33%)	
Pass	35/37 (95%)	6/6 (100%)	7/7 (100%)	13/14 (93%)	7/7 (100%)	2/3 (67%)	
T:R threshold 1:1 (1)							
Pass	37/37 (100%)	6/6 (100%)	7/7 (100%)	14/14 (100%)	7/7 (100%)	3/3 (100%)	

^1^*n*/*N* (%). ^2^Fisher’s exact test

**Table 4:** S-ICD screening success rates at T:R of 1:3 for each vector separately

Threshold/subgroup	Overall, *N* = 37^1^	ACHD, *N* = 6^1^	HCM, *N* = 7^1^	HF, *N* = 14^1^	Normal, *N* = 7^1^	S-ICD, *N* = 3^1^	*p*-value^2^
T:R threshold 1:3 (0.33) primary vector							0.34
Fail	22/37 (59%)	4/6 (67%)	4/7 (57%)	9/14 (64%)	2/7 (29%)	3/3 (100%)	
Pass	15/37 (41%)	2/6 (33%)	3/7 (43%)	5/14 (36%)	5/7 (71%)	0/3 (0%)	
T:R threshold 1:3 (0.33) alternate vector							0.95
Fail	31/37 (84%)	5/6 (83%)	5/7 (71%)	12/14 (86%)	6/7 (86%)	3/3 (100%)	
Pass	6/37 (16%)	1/6 (17%)	2/7 (29%)	2/14 (14%)	1/7 (14%)	0/3 (0%)	
T:R threshold 1:3 (0.33) secondary vector							0.1
Fail	20/31 (65%)	6/6 (100%)	3/7 (43%)	5/8 (62%)	3/7 (43%)	3/3 (100%)	
Pass	11/31 (35%)	0/6 (0%)	4/7 (57%)	3/8 (38%)	4/7 (57%)	0/3 (0%)	

^1^*n*/*N* (%). ^2^Fisher’s exact test

**Table 5:** S-ICD screening success rates at T:R of 1:2 for each vector separately

Threshold/subgroup	Overall, *N* = 37^1^	ACHD, *N* = 6^1^	HCM, *N* = 7^1^	HF, *N* = 14^1^	Normal, *N* = 7^1^	S-ICD, *N* = 3^1^	*p*-value^2^
T:R threshold 1:2 (0.5) primary vector							0.3
Fail	17/37 (46%)	3/6 (50%)	2/7 (29%)	7/14 (50%)	2/7 (29%)	3/3 (100%)	
Pass	20/37 (54%)	3/6 (50%)	5/7 (71%)	7/14 (50%)	5/7 (71%)	0/3 (0%)	
T:R threshold 1:2 (0.5) alternate vector							0.29
Fail	20/37 (54%)	3/6 (50%)	3/7 (43%)	9/14 (64%)	2/7 (29%)	3/3 (100%)	
Pass	17/37 (46%)	3/6 (50%)	4/7 (57%)	5/14 (36%)	5/7 (71%)	0/3 (0%)	
T:R threshold 1:2 (0.5) secondary vector							0.091
Fail	14/31 (45%)	4/6 (67%)	1/7 (14%)	4/8 (50%)	2/7 (29%)	3/3 (100%)	
Pass	17/31 (55%)	2/6 (33%)	6/7 (86%)	4/8 (50%)	5/7 (71%)	0/3 (0%)	

^1^*n*/*N* (%). ^2^Fisher’s exact test

**Table 6:** S-ICD screening success rates at T:R of 2:3 for each vector separately

Threshold/subgroup	Overall, *N* = 37^1^	ACHD, *N* = 6^1^	HCM, *N* = 7^1^	HF, *N* = 14^1^	Normal, *N* = 7^1^	S-ICD, *N* = 3^1^	*p*-value^2^
T:R threshold 2:3 (0.66) primary vector							0.11
Fail	12/37 (32%)	2/6 (33%)	1/7 (14%)	5/14 (36%)	1/7 (14%)	3/3 (100%)	
Pass	25/37 (68%)	4/6 (67%)	6/7 (86%)	9/14 (64%)	6/7 (86%)	0/3 (0%)	
T:R threshold 2:3 (0.66) alternate vector							0.88
Fail	13/37 (35%)	2/6 (33%)	2/7 (29%)	5/14 (36%)	2/7 (29%)	2/3 (67%)	
Pass	24/37 (65%)	4/6 (67%)	5/7 (71%)	9/14 (64%)	5/7 (71%)	1/3 (33%)	
T:R threshold 2:3 (0.66) secondary vector							< 0.001
Fail	5/31 (16%)	0/6 (0%)	0/7 (0%)	2/8 (25%)	0/7 (0%)	3/3 (100%)	
Pass	26/31 (84%)	6/6 (100%)	7/7 (100%)	6/8 (75%)	7/7 (100%)	0/3 (0%)	

^1^*n*/*N* (%). ^2^Fisher’s exact test

**Table 7:** S-ICD screening success rates at T:R of 3:4 for each vector separately

Threshold/subgroup	Overall, *N* = 37^1^	ACHD, *N* = 6^1^	HCM, *N* = 7^1^	HF, *N* = 14^1^	Normal, *N* = 7^1^	S-ICD, *N* = 3^1^	*p*-value^2^
T:R threshold 3:4 (0.75) primary vector							0.39
Fail	9/37 (24%)	2/6 (33%)	0/7 (0%)	5/14 (36%)	1/7 (14%)	1/3 (33%)	
Pass	28/37 (76%)	4/6 (67%)	7/7 (100%)	9/14 (64%)	6/7 (86%)	2/3 (67%)	
T:R threshold 3:4 (0.75) alternate vector							0.17
Fail	8/37 (22%)	2/6 (33%)	0/7 (0%)	3/14 (21%)	1/7 (14%)	2/3 (67%)	
Pass	29/37 (78%)	4/6 (67%)	7/7 (100%)	11/14 (79%)	6/7 (86%)	1/3 (33%)	
T:R threshold 3:4 (0.75) secondary vector							< 0.001
Fail	4/31 (13%)	0/6 (0%)	0/7 (0%)	1/8 (12%)	0/7 (0%)	3/3 (100%)	
Pass	27/31 (87%)	6/6 (100%)	7/7 (100%)	7/8 (88%)	7/7 (100%)	0/3 (0%)	

^1^*n*/*N* (%). ^2^Fisher’s exact test

**Table 8:** S-ICD screening success rates at T:R of 1:1 for each vector separately

Threshold/subgroup	Overall, *N* = 37^1^	ACHD, *N* = 6^1^	HCM, *N* = 7^1^	HF, *N* = 14^1^	Normal, *N* = 7^1^	S-ICD, *N* = 3^1^	*p*-value^2^
T:R threshold 1:1 primary vector							0.37
Fail	2/37 (5.4%)	0/6 (0%)	0/7 (0%)	1/14 (7.1%)	0/7 (0%)	1/3 (33%)	
Pass	35/37 (95%)	6/6 (100%)	7/7 (100%)	13/14 (93%)	7/7 (100%)	2/3 (67%)	
T:R threshold 1:1 alternate vector							0.054
Fail	2/37 (5.4%)	1/6 (17%)	0/7 (0%)	0/14 (0%)	0/7 (0%)	1/3 (33%)	
Pass	35/37 (95%)	5/6 (83%)	7/7 (100%)	14/14 (100%)	7/7 (100%)	2/3 (67%)	
T:R threshold 1:1 secondary vector							> 0.99
Fail	1/31 (3.2%)	0/6 (0%)	0/7 (0%)	1/8 (12%)	0/7 (0%)	0/3 (0%)	
Pass	30/31 (97%)	6/6 (100%)	7/7 (100%)	7/8 (88%)	7/7 (100%)	3/3 (100%)	

^1^*n*/*N* (%).^2^Fisher’s exact test

## Discussion

### Mechanism of action of S-ICD

The sensing mechanism of the S-ICD has been shown to be equally effective to the TV-ICD systems [16]. The problem arises when the device’s declining sensitivity level is met with a T-wave with an amplitude that is high enough to be inadvertently sensed by the device and misinterpreted as the next R wave (double counting). This double counting can either be silent if it did not persist long enough to trigger treating algorithms by the device or, if it persists, it can end up initiating a cascade of events that might terminate in the delivery of inappropriate shock(s).

Hypothetically, if the S-ICD did employ fixed sensitivity levels, that would mean that, for a T-wave to be sensed by the device triggering TWO, it has to have an equal amplitude to its preceding R wave (in other words a T:R ratio of 1:1), while smaller ratios should not cause any issues of oversensing provided they are consistent. S-ICDs do not employ fixed but, rather, gradually declining sensitivity levels. This means that, in order to avoid TWO, T-waves must be of an amplitude that is small enough to evade the declining sensitivity levels of their preceding R waves while also allowing for fluctuations of R and T-wave amplitudes (safety margin).

### Current S-ICD screening process

Surface ECGs of few seconds of duration done in multiple postures are used as surrogates for the three standard S-ICD vectors. These are assessed via an automated screening tool built-into an S-ICD programmer. The programmer has external ECG cables which can acquire ECG signal via the application of skin electrodes which are placed on the chest wall to capture the three S-ICD vectors and can perform surface ECG analysis allowing vector eligibility to be automatically determined. At least one vector needs to pass screening in at least two postural positions for the patient to be deemed eligible. A major predictor of eligibility of a vector is the T:R ratio where a ratio of 1:3 is currently used as a cut-off for S-ICD eligibility following the same principles as the otherwise outdated “overlay” technique.

### S-ICD Screening eligibility and inappropriate shocks rates

Approximately 5.7% of ICD recipients have no suitable S-ICD sensing vector and are therefore ineligible for an S-ICD utilising the current screening practices. This value has been derived from two separate studies in which S-ICD screening was performed on consecutive ICD patients with no pacing indication. Randles et al. reported that 3.6% of their cohort of 196 ICD patients did not have at least one S-ICD lead fulfilling surface ECG screening criteria. Nordkamp et al. reported that 7.4% of their study cohort of 230 consecutive ICD patients were considered not suitable for S-ICD due to having unfavourable surface ECGs [17, 18].

Information regarding suitability for S-ICD implantation in patients with congenital heart disease is scarce and variable. Alonso et al. conducted a study to test S-ICD eligibility specifically in congenital heart disease patients at high risk of sudden cardiac death and only 44% of the significant risk subgroup were eligible for an S-ICD [19]. In another study by Wang et al., 101 mostly complex congenital heart disease patients were screened for eligibility for S-ICD and only 61 patients (60%) were deemed eligible for S-ICD screening [20]. In another study by Garside et al., 102 complex ACHD patients were recruited and 75.4% of ACHD patients met the screening criteria for an S-ICD [21]. All these studies demonstrated higher ineligibility rates in the ACHD population than in the general population. This may be due to abnormal T-wave morphology resulting from structural and functional disturbances that characterises ACHD.

Previous studies have suggested that HCM is an independent risk factor for S-ICD screening failure [22]. Studies looking specifically into the proportion of HCM patients who satisfy the S-ICD screening criteria also reported highly varying eligibility rates. Francia et al. reported eligibility rates of 85 — 93% [23]. Maurizi et al. screened 165 HCM patients and reported a 16% screening failure rate while 36% of the high risk patients failed screening [22]. Lambiase et al. assessed 131 HCM patients with risk factors for sudden cardiac death (SCD) for eligibility for S-ICD and 38% patients were ineligible for the S-ICD with 1-vector safety and 71% were ineligible with ≥ 2-vector safety [24]. In all those mentioned studies, high T-wave voltages were the main cause of screening failure.

Despite the screening process, the main issue with the S-ICD to this date remains the relatively high rate of inappropriate shocks when compared with conventional TV-ICDs. Indeed, the most common cause of inappropriate shocks in the S-ICD population remains TWO [25–30]. T-wave morphology is in fact dynamic and can alter with position, exercise, electrolyte disturbance, progression of myocardial diseases, and changes in autonomic function. This is important as inappropriate shock therapies can have detrimental effects on the quality of life, the psychological wellbeing, and can even result in the induction of ventricular arrhythmias [31].

### Role of prolonged screening

The authors of this paper have previously introduced the concept of the potential varying of S-ICD vectors eligibility over time in a recently published study by Wiles et al. which has demonstrated that the vector score which determines S-ICD eligibility is in fact dynamic in real-life ICD population. For that study, an S-ICD simulator provided by the device manufacturer was utilised for vector assessment [32]. The clinical significance for this dynamicity is not clear, but it sheds the light on the possibility that acquiring screening data over a much longer period than for conventional screening across the three S-ICD vectors can enable more reliable and descriptive screening of the vectors and can aid patient and vector selection in S-ICD candidates.

#### Optimal T:R ratio for prolonged screening

We can see from our results that, if the “standard” T:R of 1:3 was adopted for prolonged screening, 46% of this study’s mixed cohort of patients would have been denied S-ICD therapy if they developed a clinical indication for defibrillation protection and would have likely ended up having a traditional transvenous ICD instead, with all the risks and potential complications that are associated with this. This 46% failure rate is significantly higher than the S-ICD screening failure rates reported in literature using current screening methodology. It is particularly important to point out that all of our 37 patients included in our study had a significant proportion of time of the 24-h recordings (average 96%) when they had at least one lead/S-ICD vector fulfilling the 1:3 ratio. In other words, on average, each patient in the study, regardless of underlying aetiology, had a 96% chance of passing the traditional screening methodology if they turned out on a random time of the day (24 h) for their screening. This highlights the significant discrepancy in the S-ICD screening pass rates between the current screening process and the proposed method of prolonged screening if the same T:R threshold (1:3) was adopted.

Theoretically, there is no point of examining any T:R ratios that are greater than 1:1. If a T:R ratio of 1:1 was adopted as the least strict T:R threshold theoretically feasible even with prolonged screening, all of the patients in the study regardless of their underlying aetiology would have passed the screening. A T:R of 1:1 would have been theoretically utilised if the S-ICDs employed fixed sensitivity levels, which they do not.

This puts the highest threshold (least strict) of T:R to be considered for the proposed prolonged screening methodology at just below 1:1. Adopting T:R of 1:3 as a reference point was considered because this is the threshold used for current screening practices and it showed in the results that, if this threshold was adopted, 46% of the patients cohort in the study would have not only been at risk of TWO but would have theoretically have had inappropriate shock(s) by their S-ICD devices potentially within 24 h of having their S-ICDs implanted (if each of the 24-h recordings could be considered as a representative of S-ICD vectors signals on an average day). This rate is too high and very far from the inappropriate shock rates due to TWO that occur in real life and are reported by several studies. T:R of 1:3 has proven to be too strict for prolonged screening and would likely inappropriately exclude otherwise S-ICD eligible candidates.

**Fig. 3:**
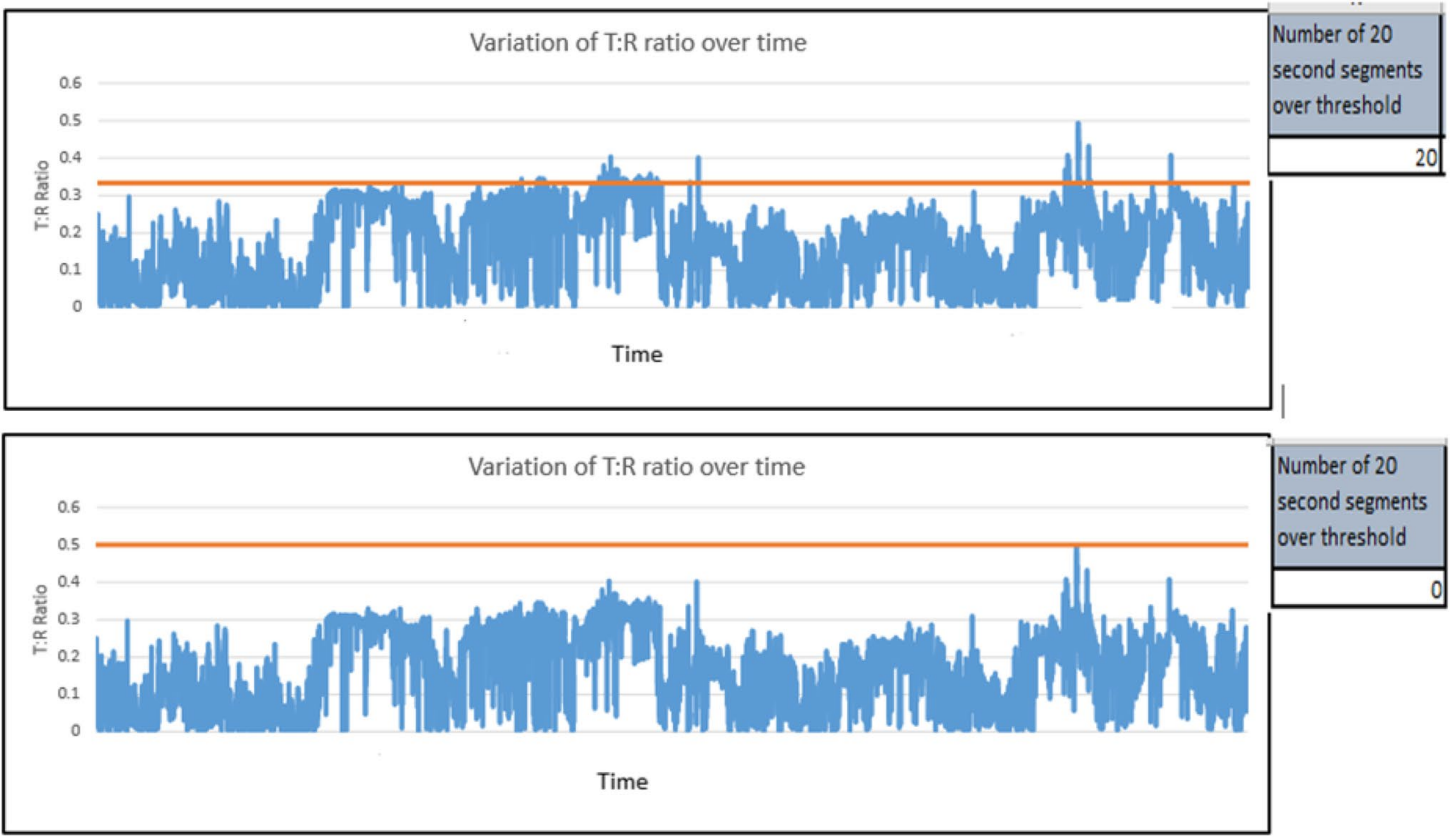
Demonstration on how slightly changing the threshold can impact the outcome of S-ICD screening. It shows the results of a recorded 24-h ECG signal corresponding to the alternate vector of an S-ICD from one of the patients from the HCM subgroup in our study as analysed by our tool. There is a clear demonstration of T:R ratio fluctuation along the recorded 24 h. The top half of the figure shows how the T:R ratio crosses the proposed T:R threshold of 1/3 on 20 occasions convincingly failing the screening, while the bottom half shows how the T:R ratio never crossed the proposed T:R threshold of 1/2, effectively passing the screening if we choose to adopt this threshold

In our study, experimentation with a range of T:R ratios ranging between the (standard) 1:3 up to the (least strict but theoretically feasible) 1:1 was done, demonstrating how slightly changing the T:R ratio cut-off for eligibility can have a huge impact on the S-ICD eligibility rates specifically for each cohort of patients as well as on the overall eligibility (Fig. 3). The aim of this study was to introduce the concept of prolonged screening for S-ICD eligibility utilising machine learning methods and the need to revisit the current screening parameters if this is to be adopted. A prospective study with real-life S-ICD candidates and long-term follow-up is needed to give insight on the optimal T:R ratio(s) that could be utilised for prolonged screening for S-ICD eligibility.

## Limitations

There are several limitations to our study. First of all, our study focuses on the T:R ratio as the major determinant of S-ICD eligibility, not counting any other parameters which can contribute to the passing or failing of the S-ICD screening. Secondly, the number of the patients recruited in this study is small; as such, results need to be interpreted with caution and larger adequately powered studies are needed to consolidate our findings. In addition, the average heart rates were not incorporated when rejecting a pre-defined ratio, this could be relevant as double counting at a low heart rate is less likely to trigger inappropriate shock therapy than that at a higher rate. It is likely that for future studies, the tool could be adjusted to incorporate this parameter. Moreover, our proposed methodology also does not consider relatively newer algorithms such as SMART PASS that are integrated into the S-ICD that can help it differentiate between R and T-waves based on other characteristics rather than just their amplitudes, and potential further work could incorporate new software such as smart pass into our deep learning tool. By choosing a consecutive 20-s periods as cut-off for screening, the time needed for the device to deliver a shock is effectively ignoring other potential clinically silent oversensing episodes, allowing those vectors to theoretically pass the screening despite the “silent” oversensing episodes. However, our tool can be adjusted for both the cut off thresholds as well as the time intervals to adapt the screening thresholds as needed. Also, our proposed T:R cut-offs are arbitrary but, as we mentioned earlier, our aim was not to pinpoint the optimal T:R ratio but, rather, to introduce the concept of prolonged screening and the potential need to revisit the S-ICD screening criteria. Finally, the clinical relevance of our findings is not clear, while the variations in the T:R ratios that is demonstrated in our study is theoretically significant, and we do not know for certain that it necessarily translates into adverse clinical events. Further experimental work is needed to consolidate our findings and to incorporate them into clinical practice.

## Conclusion

We have proposed a new methodology adopting artificial intelligence and deep learning methods that can theoretically help in patient selection for S-ICD therapy while minimising the risk of inappropriate shocks due to cardiac oversensing. The clinical implications of our findings for both screening and for long-term sensing assurance is yet to be established. Whether our proposed methodology, can in fact reduce TWO in a real-world scenario, with the limitations of having fixed S-ICD sensing vector programming, is yet to be defined. Further work is required, and adequately powered studies are needed to identify the optimal screening thresholds before this can be translated into clinical practice.
